# Analysis of SARS-CoV-2 Genomes from West Java, Indonesia

**DOI:** 10.3390/v13102097

**Published:** 2021-10-18

**Authors:** Azzania Fibriani, Rebecca Stephanie, Afifah Alifia Alfiantie, Agust Leo Fany Siregar, Gusti Ayu Prani Pradani, Nicholas Yamahoki, William Steflandel Purba, Cut Nur Cinthia Alamanda, Ema Rahmawati, Rifky Waluyajati Rachman, Rini Robiani, Ryan Bayusantika Ristandi

**Affiliations:** 1 Bandung Institute of Technology, Bandung, West Java 40132, Indonesia; rebecca.stephanie@students.itb.ac.id (R.S.); ifahalifia@students.itb.ac.id (A.A.A.); leoagust357@students.itb.ac.id (A.L.F.S.); gustiayuprani@students.itb.ac.id (G.A.P.P.); yamahoki@students.itb.ac.id (N.Y.); williamsteflandel@students.itb.ac.id (W.S.P.); 2 West Java Health Laboratory, Bandung, West Java 40161, Indonesia; cut_alamanda@yahoo.co.id (C.N.C.A.); Emmakuda72@gmail.com (E.R.); rifky26@gmail.com (R.W.R.); rinirobiani66@gmail.com (R.R.); ryanryudavinaria@gmail.com (R.B.R.)

**Keywords:** Indonesia, mutation, protein stability, SARS-CoV-2, variant, West Java

## Abstract

West Java Health Laboratory (WJHL) is one of the many institutions in Indonesia that have sequenced SARS-CoV-2 genome. Although having submitted a large number of sequences since September 2020, however, these submitted data lack advanced analyses. Therefore, in this study, we analyze the variant distribution, hotspot mutation, and its impact on protein structure and function of SARS-CoV-2 from the collected samples from WJHL. As many as one hundred sixty-three SARS-CoV-2 genome sequences submitted by West Java Health Laboratory (WJHL), with collection dates between September 2020 and June 2021, were retrieved from GISAID. Subsequently, the frequency and distribution of non-synonymous mutations across different cities and regencies from these samples were analyzed. The effect of the most prevalent mutations from dominant variants on the stability of their corresponding proteins was examined. The samples mostly consisted of people of working-age, and were distributed between female and male equally. All of the sample sequences showed varying levels of diversity, especially samples from West Bandung which carried the highest diversity. Dominant variants are the VOC B.1.617.2 (Delta) variant, B.1.466.2 variant, and B.1.470 variant. The genomic regions with the highest number of mutations are the spike, NSP3, nucleocapsid, NSP12, and ORF3a protein. Mutation analysis showed that mutations in structural protein might increase the stability of the protein. Oppositely, mutations in non-structural protein might lead to a decrease in protein stability. However, further research to study the impact of mutations on the function of SARS-CoV-2 proteins are required.

## 1. Introduction

Since its first appearance in Wuhan, China, at the end of 2019, the coronavirus disease (COVID-19), caused by SARS-CoV-2, is rapidly spreading globally. In Indonesia, the first identified case of COVID-19 was reported in early March 2020; and by March 2021 COVID-19 has amounted to 1.3 million cases, and was responsible for 36,000 deaths [1]. With these numbers, Indonesia was considered as the top 20 countries with the most COVID-19 cases in the world, and was the first in Southeast Asia. Among Indonesian government efforts to handle these problems were massive surveillance for new COVID-19 cases and implementation of COVID-19 vaccination programs across the country [1].

As Indonesia continued to battle this issue, SARS-CoV-2 also found its way to sustain through constant mutations that eventually lead to the formation of multiple new variants. Mutation or a change of nucleotide in genes could cause a change in protein structure and function. Moreover, mutations could also cause a change in viral characteristics, potentially leading to an outbreak, reduce vaccine effectiveness, and set back the development of antiviral and diagnostic kits [2]. Mutations in SARS-CoV-2 that had been reported to reduce antibody neutralization were found in variant B.1.1.7 and B.1.35; this finding could entail the futility of the vaccination program that was done in the hope of acquiring herd immunity [3,4].

The viral genome provides various information ranging from viral characteristics, pathogenesis, origin, transmission, mutation profile, and diversity of viral variant. A mutation followed by high prevalence describes the diversity of variants in a given population within a time. Increasing the frequency of any mutation can indicate the emergence of a new variant with different characteristics, thus changing the pattern of the diversity variant in that area [5].

Since 2020 many institutions in Indonesia have sequenced SARS-CoV-2 viruses using the whole-genome sequencing (WGS) method, one of them being West Java Health Laboratory (WJHL). WJHL first submitted their SARS-CoV-2 sequence in September 2020, and has continued to do so to this day. This can serve to support SARS-CoV-2 treatment guidelines in Indonesia or prepare for new variants in the future by giving a scientific perspective, but comprehensive analyses on these sequences are still scarce. We analyzed SARS-CoV-2 sequenced by WJHL between September 2020 and June 2021, including variant distribution, hotspot mutation, and its impact on protein structure and function of SARS-CoV-2.

## 2. Materials and Methods

### 2.1. Data Collection

One hundred sixty-three SARS-CoV-2 genome sequences submitted by West Java Health Laboratory (WJHL), collected between September 2020 and June 2021, were downloaded from the GISAID database [6]. Genome sequences with a size of more than 29 kb (complete genome), high coverage (with <1% Ns), and collection date information are included in the analysis. Metadata of the sequences includes information of sequence origin, collection date, sampling strategy, specimen type, patient gender, age, and status. Sequence accession IDs, sequencing technology, and assembly methods are available in Supplementary Table S1.

### 2.2. Analysis of Distribution of Variants and Mutations

SARS-CoV-2 genome sequences from different cities and regencies were grouped by their variants. Mutations were analyzed using tools available on CoV-GLUE mutation database [7], with severe acute respiratory syndrome coronavirus 2 isolate Wuhan-Hu-1 (NC_045512.2) as a reference. The analysis focused on non-synonymous mutations, including substitutions, deletions, and insertions. The occurrence frequency of each mutation is calculated by dividing the number of sequence samples carrying the mutation by the total number of samples analyzed. Mutations with a frequency of more than 10% are considered hotspot mutations [5]. Statistical analysis was performed using Microsoft Excel version 2013 and IBM SPSS Statistics for Windows version 24.0. Classification of SARS-CoV-2 follows the PANGO lineages.

### 2.3. Analysis of Protein Stabilization

Three-dimensional structure of the spike, NSP12, NSP13, and ORF7a protein was obtained from online protein databank (www.rcsb.org/, accessed on: 1 July 2021), with PDB ID 7A29, 6NUR, 6ZSL, and 7CI3, respectively. Meanwhile, the 3D structure of the nucleocapsid, membrane, NSP3, and ORF3a protein was modeled using i-TASSER online web-server (https://zhanggroup.org/COVID-19/, accessed on: 20 June 2021) [8]. The ΔΔG value (kcal/mol) (ΔΔG = ΔGmutant − ΔGwildtype) of each protein due to mutation was calculated using FoldX version 5.0 plugin in YASARA, with one time run at temperature 310 K. A value of ΔΔG < −0.5 shows a stabilizing effect on the protein, destabilizing for a value of ΔΔG > 0.5, and neutral for −0.5 < ΔΔG < 0.5 (no significant change in protein stability) [9,10]. Analysis was performed for mutations appearing in all sequences for each variant, and appearing in genomic regions constituting a large proportion of mutations across all variants, unless indicated otherwise.

### 2.4. Molecular Docking

Protein docking analysis between the protein of genomic region with the highest number of mutations across dominant SARS-CoV-2 variants and its substrate, which had previously been prepared, was performed using HADDOCK version 2.4 online web-server (https://wenmr.science.uu.nl/haddock2.4/, accessed on 27 July 2021) with default parameters [11]. The binding affinity (kcal/mol) of the protein was calculated by PRODIGY web-server (https://wenmr.science.uu.nl/prodigy/, accessed on 29 July 2021) [12]. PyMol software was used to visualize the 3D structure of the protein complex [13].

## 3. Results and Discussions

**Figure 1:**
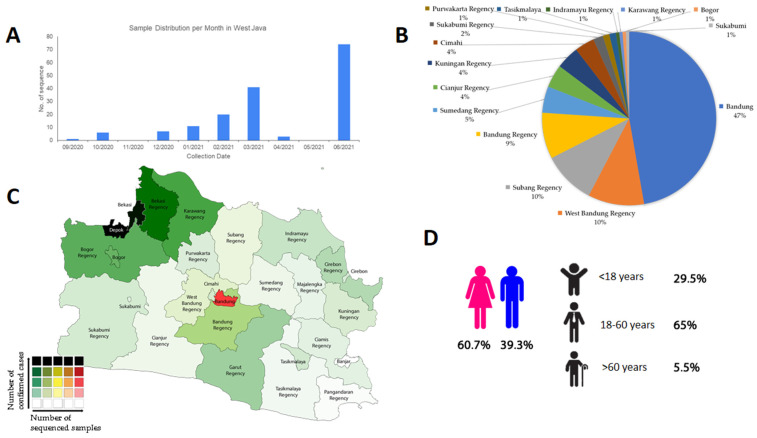
Summary of the epidemiology of SARS-CoV-2 samples sequenced by WJHL, isolated from West Java, Indonesia. (**A**) Time series of SARS-CoV-2 samples collection (*n* = 163). (**B**) Proportion of SARS-CoV-2 samples across cities and regencies in West Java, Indonesia. (**C**) Distribution of SARS-CoV-2 sequenced samples across different cities and regencies in West Java, Indonesia. (**D**) Demography of samples.

The number of SARS-CoV-2 samples from September 2020 to June 2021 showed a significant increase, although decreases were documented briefly in November 2020, April 2021, and May 2021, as shown in Figure 1A. Bandung contributed most of the samples (47%), followed by West Bandung (10%), Subang (10%), Bandung Regency (9%), and Sumedang (5%), as shown in Figure 1B,C. Most of our samples were female patients (60.7%) and working-age patients (age 18 through 60, accounting for 65% of the samples), as shown in Figure 1D. This is consistent with other findings in Southeast Asia and Asia, where most positive COVID-19 cases are dominated by people of working age with no distinct segregation between females and males [14,15]. In Indonesia, sequenced samples were comprised of 50.5% female samples and 48.6% male samples (with 0.9% of the samples having no information). In Southeast Asia and Asia, a significantly large proportion of the samples with no information on sex hindered the female-to-male proportion from appearing as 50-50, but still showed a ratio close to 1-1 [16].

**Figure 2:**
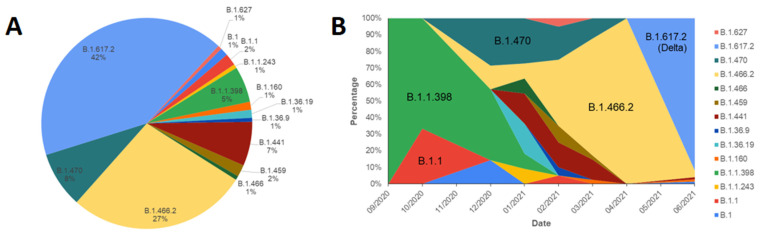
Distribution of SARS-CoV-2 variants from September 2020 to June 2021. (**A**) Proportion of variants. (**B**) Time series of SARS-CoV-2 for each variant. VOC B.1.617.2 (Delta) and two local Indonesian variants (B.1.466.2 and B.1.470) dominated West Java. The shifts of different variants were observed over time.

Shown in Figure 2A, samples from September 2020 to June 2021 belonged to the B PANGO variant, furthermore dominated by the VOC B.1.617.2 (Delta) variant (42%), B.1.466.2 variant (27%), and B.1.470 variant (8%), the last two enlisted as Indonesian lineages by PANGO although not being a variant of concern at a global level [17]. B.1.466.2 has also been designated Alerts for Further Monitoring by the World Health Organization [18]. Other Indonesian lineages enlisted by PANGO, the B.1.1.398 and B.1.459 variants [17], contributed only to a small percentage in the samples, 5% and 2% respectively, while the B.50 was not found in this study [15,17]. B.1.466.2, B.1.470, B.1.1.398, and B.1.459 were more locally distributed in Indonesia and were found in lower percentages in other Southeast Asian countries [15]. The two other VOCs that are primarily known to circulate in Southeast Asia, the Alpha B.1.1.7 and Beta B.1.351 variants [19], were absent in this study, although making up for 19.2% and 1.6% of the Asian samples respectively, and 43.1% and 1.1% of global samples respectively. Indeed it was found that predominating variants in Indonesia were B.1.466.2 and B.1.470 as of 1 June 2021, and that the Alpha and Beta variants were more dominating in other regions of Southeast Asia, such as Thailand, Cambodia, Philippines, and Vietnam [19], and in Asia as a whole [15].

Plotting the PANGO variants of the samples against time (Figure 2B) showed an initial dominance of B.1.1.398 in September 2020, followed by the identification of B.1.1, B.1.466.2, and B.1.470 in the following month. Variants B.1.1.398 and B.1.470 were observed to dominate samples by December 2020 before the emergence of B.1.466.2, dominating from January through April 2021. The Delta variant first appeared in West Java on April 2021 [20] and has since increased before dominating other variants in WJHL samples. This phenomenon is consistent with the pattern observed in West Java and Indonesia [21]. The Delta variant is known to have been first documented considerably early during the pandemic, in India on September 2020, before appearing in Indonesia in Jakarta in January 2021, but had only been designated Variant of Concern by the World Health Organization in 11 May 2021 [17,18]. During the third week of August 2021 alone, 47.4% of all the new cases nationwide reported by the Indonesian Ministry of Health were of this variant, contrasting the Alpha variant that only constituted 0.003% of all the new reported cases and the absence of new Beta cases. The other two Indonesian lineages enlisted by PANGO, B.1.466.2 and B.1.470, originated from Indonesia and were first documented in November 2020 and March 2020 respectively [17]. During the third week of August 2021, B.1.466.2 and B.1.470 were reported to contribute to 31.7% and 0.03% of all the cases in the country [22].

**Figure 3:**
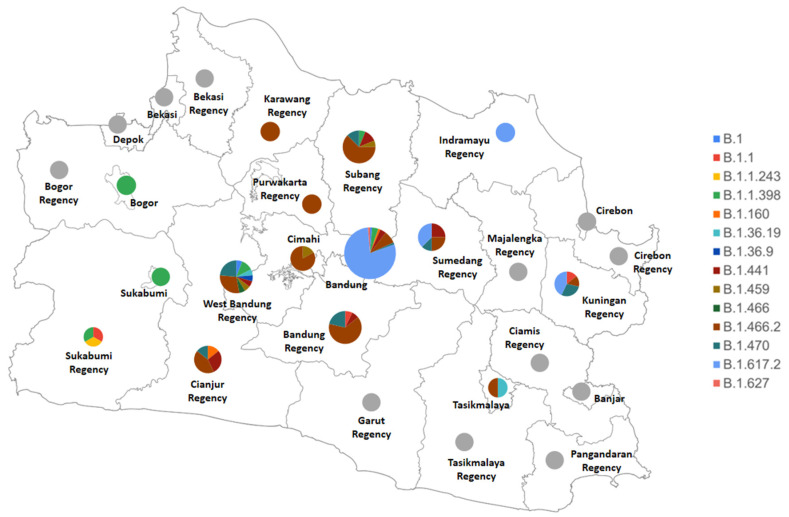
Map of diversity of SARS-CoV-2 variants circulating in each city and district of West Java, Indonesia in September 2020–June 2021. Grey color indicates absence of samples from the city/regency.

West Java is observed to house a variety of dominating PANGO variants across its cities and regencies, as shown below in Figure 3. Regions with the highest diversity in variants are West Bandung Regency (carrying 9 variants, dominated by B.1.466.2 and B.1.470), Bandung (carrying 8 variants, dominated by the Delta variant, B.1.617.2), and Subang Regency (carrying 5 variants, dominated by B.1.466.2). Several variants only appeared in particular regions, such as the B.1.1.243 (appearing once in Sukabumi Regency), B.1.36.9 and B.1.466 (both only appearing once in West Bandung Regency), and B.1.627 (appearing once in Bandung).

The high diversity of SARS-CoV-2 variants in West Bandung Regency might serve as a point of interest, considering how Bandung showed a lower diversity although being the capital of West Java. Beside B.1.466.2 and B.1.470 that generally dominated Indonesia at a national level [22], West Bandung Regency was also found to house the other Indonesian lineages (B.1.1.398 and B.1.459), and other minor variants such as the B.1, B.1.36.19, B.1.36.9, B.1.441, and B.1.466 variants. These minor variants in West Bandung Regency were also found in different parts of the world, with the B.1.441 and B.1.466 variants even enlisted as global lineages by PANGO [17]. This might be due to West Bandung Regency being the major homecoming destination for a great number of West Java citizens, where other variants from other places in the province might be carried by the large wave of people coming home for the Eid al-Fitr. Indonesia is a Muslim-majority country, and homecoming has become a tradition for Indonesian Muslims to celebrate, at the end of Eid Al-Fitr. Moreover, West Bandung Regency is also known for its recreational sites, which, despite the continuously increasing number of positive cases during the month of June, were still operating although with health safety measures being enforced [20]. Following the first community activities restrictions (PPKM) in Indonesia on January 2021 [23], WHO Indonesia reported that retail and recreational activities as well as transit stations activities in West Java were maintaining their numbers, if not increasing in trend [24]. This finding suggests a relatively great number of people circulating in West Bandung Regency right before the period where positive cases were continuously increasing in this area. It might propose an appealing point that could be further explored in later studies: the possibility of within-host co-infection and recombination by multiple SARS-CoV-2 variants that might contribute to the high diversity of SARS-CoV-2 variant in West Bandung Regency.

Furthermore, although this increase in citizens mobility can also be observed in other provinces with major cities in Indonesia (such as Jakarta, Banten, and Central Java), unfortunately, reports of SARS-CoV-2 diversity in Indonesia only calculate the variants of concern, thus making it difficult to examine all SARS-CoV-2 variants across provinces in Indonesia [22,24,25].

It should also be noted that some regions may appear to only house one or several particular variants not because of the absolute absence of other variants, but rather because of the small sample size that might not entirely reflect the dynamics of the variants in these regions.

**Figure 4:**
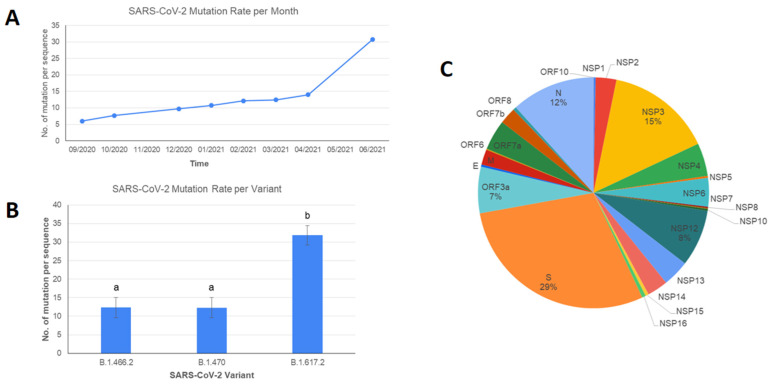
Distribution of mutations in SARS-CoV-2 samples sequenced by WJHL, isolated from West Java, Indonesia from September 2020 to June 2021. (**A**) Mutation rate per month. (**B**) Mutation rate for SARS-CoV-2 variants dominating WJHL samples. Error bars represent standard deviations of the means. Different letters on top of each bar show a significant difference between variants according to Kruskal-Wallis test (*p* = 0.05). Kruskal-Wallis test results are available in Supplementary Figure S1. (**C**) Percentage of hotspot mutations across SARS-CoV-2 genomic regions. Percentage of each genomic region is expressed by dividing the number of all mutations found in the region by the total number of mutations found in all samples. Details on hotspot mutations are available in Supplementary Table S2.

Number of mutations of SARS-CoV-2 in this study showed a constant rate of increase from September 2020 to April 2021, followed by a significant escalation from April to June 2021 (Figure 4A). This sudden jump in mutation rate was likely caused by the introduction of the B.1.617.2 Delta variant in Indonesia. Studies comparing the mutation rate of the Delta variant to other dominant variants are still unavailable. However, the previous study showed that the spike gene of the Delta variant has a higher number of mutations compared to any other variants of concerns (Alpha, Beta, and Gamma), as shown in Figure 4B [26,27]. Over all the variants, mutations were mostly found in the spike gene (29%), NSP3 (15%), N (12%), NSP12 (8%), and ORF3a (7%). Mutation hotspots for SARS-CoV-2 found in this study were similar to those circulating globally, where the majority of the mutation found in high frequency were located in the spike, NSP12, nucleocapsid, and ORF3a, with only NSP3 as an exception as it is not considered as the gene where the majority of high-frequency mutation was located, but still a site with a high number of mutations nonetheless [28]. The highest frequency of NSP3 mutations among the Delta variant includes T678I, P1469S, P1228L, A488S. Except for P1228L, all the mutations are identified as the highest frequency of NSP3 mutations across all the countries with Delta variants [29].

**Figure 5:**
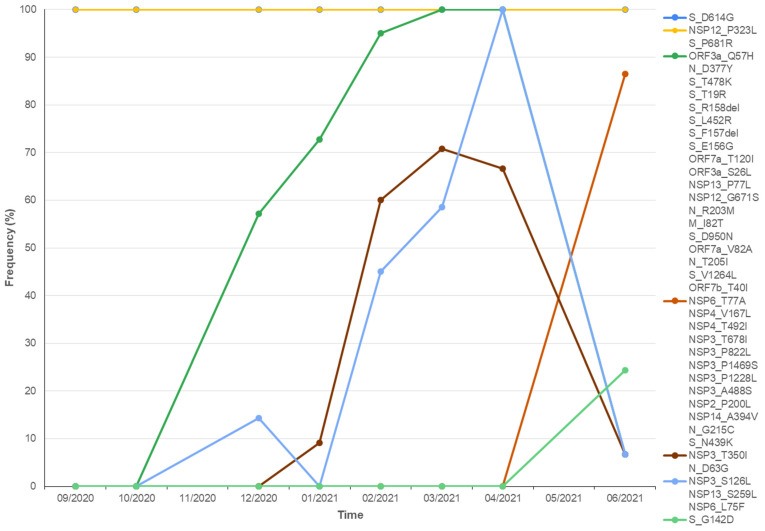
Dynamics of hotspot mutations of SARS-CoV-2 samples in this study circulating in West Java over time, from September 2020 to June 2021. The frequency of each hotspot mutation is expressed by dividing the number of the mutation per month with the number of samples in the month. Not all mutations are displayed for proper presentation.

Plotting the frequency of hotspot mutations occurrence against time showed that different mutations fluctuated over time. Figure 5 displays some examples of this phenomenon. The D614G mutation in the spike protein and the P323L mutation in the NSP12 are constantly present in all samples from September 2020 to June 2021. Q57H in the ORF3a was first documented at the end of 2020 and underwent a rise in number until April 2021. S126L in NSP3 was first documented at the end of 2020, before disappearing, making a constant rise in number since its reemergence in February 2021, peaking at April 2021, and decreasing through June 2021. T350I in NSP3 was first documented at the end of 2020, constantly increasing until March 2021 and decreasing until June 2021. G142D in the spike and T77A in the NSP6 protein were first documented in April 2021 and have since increased.

Our study analyzed the effect of the mutation in the three dominating variants on the structural stability of their corresponding proteins. Mutations in B.1.466.2 and B.1.617.2 (Delta variant) mostly stabilized structural protein (ΔΔG ≤ −0.5), while those in B.1.470 mostly have a destabilizing effect on the NSP12 and ORF3a protein (ΔΔG ≥ 0.5). Across the three dominating variants, mutations in the spike usually contribute an advantage to its structural stability, while mutations in the NSP12 all decreased the stability of this protein.

It has been shown from the analysis that mutations occurring in the structural protein generally tend to increase the protein structural stability. For instance, D614G in the spike protein found in the three dominating variants increases protein stability (details on the protein stability analysis are available in Supplementary Table S3). Spike protein is known to interact with the ACE2 receptor in humans for the viral entry process into the host [30]; thus, simulation of this interaction (spike protein PDB ID: 7A29; ACE2 protein PDB ID: 6CS2) and calculation of the binding affinity (in kcal/mol) using molecular docking analysis were performed (Supplementary Table S4). All three mutant variants were found to show stronger binding affinities than the wild-type protein (shown in Supplementary Figure S2) indicating the interaction can occur more spontaneously. In the case of variant B.1.617.2, although individual mutation has a destabilizing effect on the protein, accumulated mutations on different sites of the spike protein cause the binding affinity of the protein from B.1.617.2 with ACE2 as its receptor to be relatively stronger than the other two variants.

On the other hand, mutations spotted in non-structural proteins mostly lower the viral protein stability. NSP3, a multi-domain protein in SARS-CoV-2 including papain-like protease that assists in viral polyprotein processing or formation of replication-transcription complexes (RTCs) [31], harbors several mutations with a high prevalence for the Delta (B.1.617.2) variant. For instance, T678I, P1228L, and P1469S were observed to reduce protein stability. However, since these mutations are not located in the protease domain (aa 783–1036), it is still unclear whether these mutations negatively affect the performance of the protease. On the other hand, P822L, found in the B.1.466.2 variant and located in the protease domain, as well as A488S from the Delta variant, has insignificant effect on the protein stability. All mutations in the NSP12 protein consistently show destabilizing effect in the three dominating variants. NSP12, also known as RNA-dependent RNA polymerase (RdRp), plays a central role in the replication and transcription process in the SARS-CoV-2 virus [32]. Mutations in RdRp may influence the viral replication and transcription processes, and may even lead to the emergence of another mutation due to errors in the replication and transcription machinery. P77L mutation in the NSP13 protein also shows destabilizing effect, but this mutation only occurred in the Delta variant. Mutations found in NSP13 in this experiment show a consistent pattern of lowering the protein stability and may have detrimental effects on its function. Lastly, T120I can be observed in the ORF7a protein, but this mutation presumably should not affect the function of this protein considering the neutral ∆∆G value [33].

## 4. Conclusions

SARS-CoV-2 samples sequenced by WJHL from West Java, Indonesia, between September 2020 and June 2021 showed that these samples are mostly of the working-age, with no distinct segregation by sex. These samples originated from different cities and regencies in West Java, with varying levels of diversity. West Bandung is observed to carry the highest diversity. Nevertheless, the variants that were observed to be dominating other variants are the VOC B.1.617.2 (Delta) variant, B.1.466.2 variant, and B.1.470 variant. Across all these variants, mutations were mostly found in the spike, NSP3, nucleocapsid, NSP12, and ORF3a protein. Regarding the virus diversity, the possibility of quasispecies in SARS-CoV-2 is also an interesting point that could be further explored in later studies.

Regarding protein stability, mutations occurring in the structural protein tend to increase the protein structural stability and increase the binding affinity between the viral spike protein and human ACE2 receptor. Meanwhile, mutations in non-structural proteins mostly lower the viral protein stability. However, the impact of these mutations on the function of SARS-CoV-2 proteins is still unknown and may be illuminated in further studies in the future.

## Data Availability

Data is contained within the article and supplementary material.

## Supplementary Materials

The following are available online at https://www.mdpi.com/article/10.3390/v13102097/s1. Table S1: List of SARS-CoV-2 genome sequences from West Java Health Laboratory (WJHL) analyzed in this study. Sequences retrieved from GISAID (https://www.gisaid.org/, accessed on: 20 June 2021). Table S2: Hotspot mutations (frequency >10% of samples) found in SARS-CoV-2 genome sequences circulating in Indonesia. Mutations are sorted in descending frequency order. Table S3: Analysis of mutations found in dominant variants (B.1.466.2, B.1.470 and B.1.617.2) on protein stability. Stability change is shown as ΔGmutant − ΔGwildtype. (ΔΔG > 0.5: destabilize; ΔΔG < −0.5: stabilize; −0.5 < ΔΔG < 0.5: neutral). Mutations in grey are mutations appearing with a prevalence of higher than 90% in each variant (not appearing in all sequences). Table S4: Molecular docking analysis of spike protein (wildtype, B.1.466.2, B.1.470 and B.1.617.2 variant) with ACE2 receptor. Figure S1: Kruskal–Wallis test for the significance of mutation rate of three dominant SARS-CoV-2 variants. Mutation rate of the B.1.617.2 Delta variant is significantly higher than the other two dominant variants (tested using the Kruskal–Wallis test, with *p* < 0.05). Figure S2: Visualization of interaction between spike protein of B.1.466.2, B.1.470, and B.1.617.2 variant with ACE2 receptor along with position of mutated residues show in red circles.
