# Supplementary material for: Analysis of SARS-CoV-2 Genomes from West Java, Indonesia
Source: Viruses. 2021 Oct 18;13(10):2097. doi: 10.3390/v13102097 (PMC8538575; doi:10.3390/v13102097)
Supplement: Supplementary file 1 [file viruses-13-02097-s001.zip › viruses-1406632-supplementary.pdf]

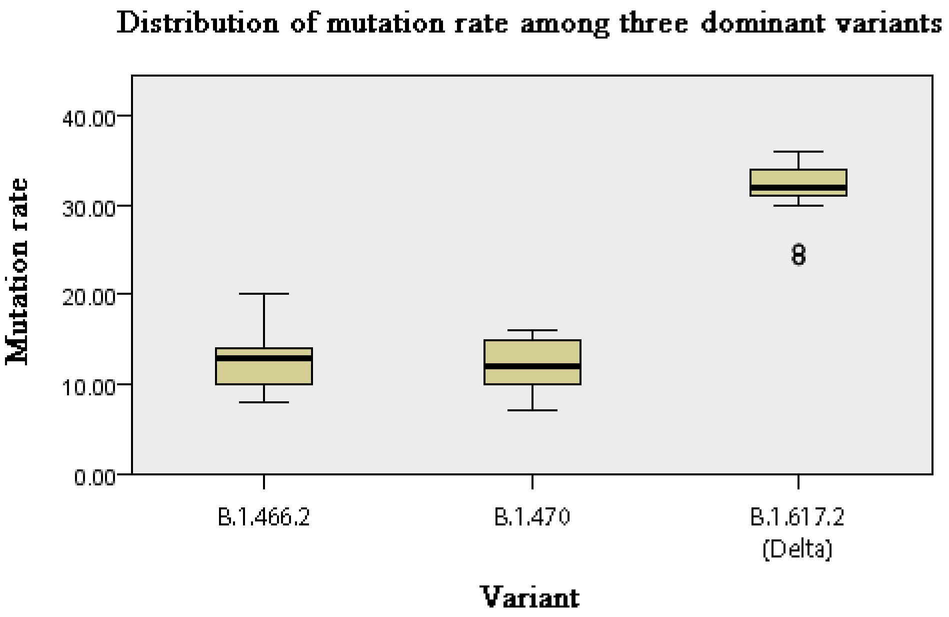

Supplementary Figure S1. Kruskal–Wallis test for the significance of mutation rate of three dominant SARS-CoV-2 variants. Mutation rate of the B.1.617.2 Delta variant is significantly higher than the other two dominant variants (tested using the Kruskal–Wallis test, with $p < 0.05$).

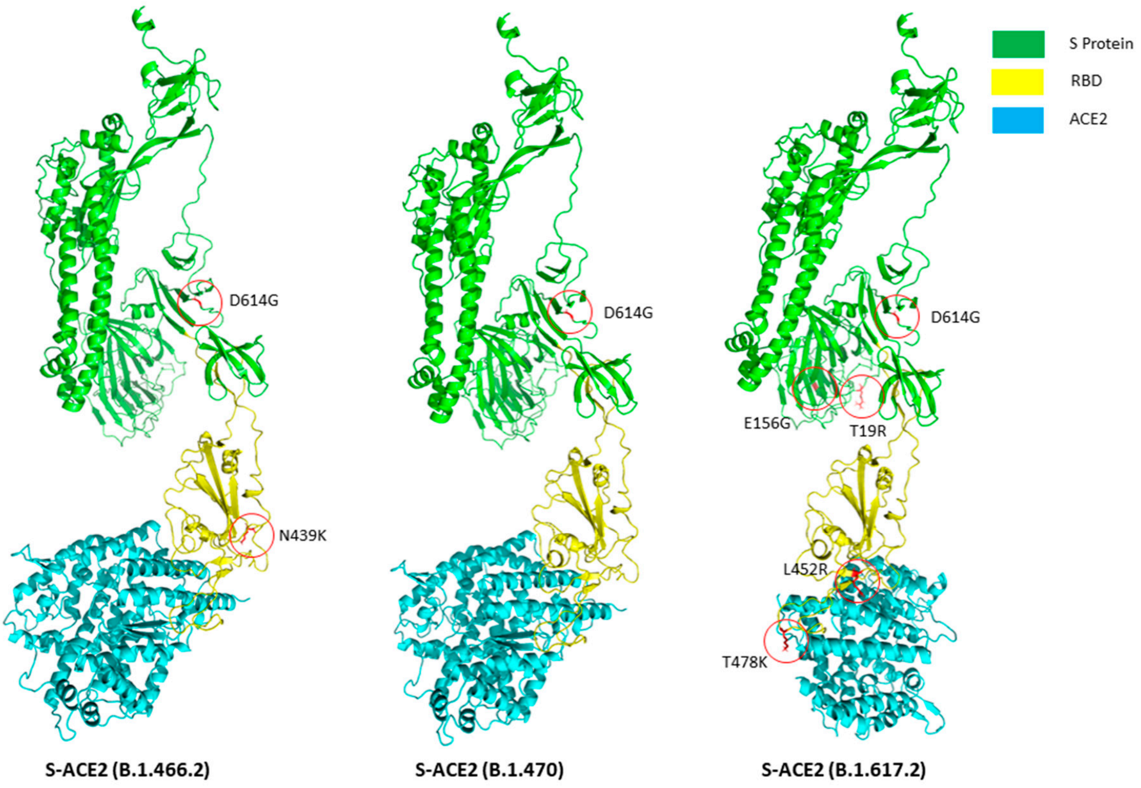

Supplementary Figure S2. Visualization of interaction between spike protein of B.1.466.2, B.1.470, and B.1.617.2 variant with ACE2 receptor along with position of mutated residues show in red circles.

Supplementary Table S1 List of SARS-CoV-2 genome sequences from West Java Health Laboratory (WJHL) analyzed in this study. Sequences retrieved from GISAID (https://www.gisaid.org/).

No.	ACCESSION ID	Sequencing technology	Assembly method
1	EPI_ISL_1340862	Illumina NextSeq	DRAGEN COVID Lineage BaseSpace
2	EPI_ISL_1340865	Illumina NextSeq 500	DRAGEN COVID Lineage BaseSpace
3	EPI_ISL_1340866	Illumina NextSeq 500	DRAGEN COVID Lineage BaseSpace
4	EPI_ISL_1340867	Illumina NextSeq 500	DRAGEN COVID Lineage BaseSpace
5	EPI_ISL_1341792	Illumina NextSeq 500	DRAGEN COVID Lineage BaseSpace
6	EPI_ISL_1341793	Illumina NextSeq 500	DRAGEN COVID Lineage BaseSpace
7	EPI_ISL_1341796	Illumina NextSeq 500	DRAGEN COVID Lineage BaseSpace
8	EPI_ISL_1341797	Illumina NextSeq 500	DRAGEN COVID Lineage BaseSpace
9	EPI_ISL_1341800	Illumina NextSeq 500	DRAGEN COVID Lineage BaseSpace
10	EPI_ISL_1341801	Nanopore GridION	RAMPART
11	EPI_ISL_1341802	Illumina MiSeq	CLC Genomics Workbench
12	EPI_ISL_1341907	Illumina MiSeq	CLC Genomics Workbench
13	EPI_ISL_1343218	Illumina MiSeq	CLC Genomics Workbench
14	EPI_ISL_1344587	Illumina MiSeq	CLC Genomics Workbench
15	EPI_ISL_1346047	Illumina MiSeq	CLC Genomics Workbench
16	EPI_ISL_1346484	Illumina NextSeq 500	DRAGEN COVID Lineage BaseSpace
17	EPI_ISL_1346625	Illumina NextSeq 500	DRAGEN COVID Lineage BaseSpace 4212x
18	EPI_ISL_1346627	Illumina NextSeq 500	DRAGEN COVID Lineage BaseSpace
19	EPI_ISL_1398267	Illumina NextSeq 500	DRAGEN COVID Lineage BaseSpace
20	EPI_ISL_1398353	Illumina NextSeq 500	DRAGEN COVID Lineage BaseSpace
21	EPI_ISL_1398355	Illumina NextSeq 500	DRAGEN COVID Lineage BaseSpace
22	EPI_ISL_1398357	Illumina NextSeq 500	DRAGEN COVID Lineage BaseSpace
23	EPI_ISL_1398359	Illumina NextSeq 500	Geneious Prime v.2021.1.1
24	EPI_ISL_1398361	Illumina NextSeq 500	DRAGEN COVID Lineage BaseSpace
25	EPI_ISL_1398363	Illumina NextSeq 500	DRAGEN COVID Lineage BaseSpace
26	EPI_ISL_1398365	Illumina MiSeq, Sanger	CLC Genomics Workbench 12
27	EPI_ISL_1398367	Illumina MiSeq, Sanger	CLC Genomics Workbench 12
28	EPI_ISL_1398369	Illumina MiSeq, Sanger	CLC Genomics Workbench 12
29	EPI_ISL_1398371	Illumina MiSeq	BaseSpace
30	EPI_ISL_1398374	Illumina MiSeq	BaseSpace
31	EPI_ISL_1398375	Illumina MiSeq	BaseSpace
32	EPI_ISL_1398561	Illumina MiSeq	BaseSpace
33	EPI_ISL_1398562	Illumina MiSeq	BaseSpace
34	EPI_ISL_1398563	Illumina MiSeq	BaseSpace
35	EPI_ISL_1398565	Illumina MiSeq	BaseSpace
36	EPI_ISL_1398567	Illumina MiSeq	BaseSpace
37	EPI_ISL_1398568	Illumina MiSeq	BaseSpace
38	EPI_ISL_1398924	Illumina MiSeq	BaseSpace

39	EPI_ISL_1398929	Illumina MiSeq	BaseSpace
40	EPI_ISL_1398930	Illumina MiSeq	CLC Genomics Workbench
41	EPI_ISL_1398932	Illumina MiSeq	CLC Genomics Workbench
42	EPI_ISL_1398934	Illumina MiSeq	CLC Genomics Workbench
43	EPI_ISL_1398936	Illumina MiSeq	CLC Genomics Workbench
44	EPI_ISL_1398939	Illumina MiSeq	CLC Genomics Workbench
45	EPI_ISL_1398942	Illumina MiSeq	CLC Genomics Workbench
46	EPI_ISL_1398955	Illumina MiSeq	CLC Genomics Workbench
47	EPI_ISL_1398980	Illumina MiSeq	CLC Genomics Workbench
48	EPI_ISL_1398981	Illumina MiSeq	CLC Genomics Workbench
49	EPI_ISL_1398983	Illumina MiSeq	CLC Genomics Workbench
50	EPI_ISL_1398984	Illumina MiSeq	CLC Genomics Workbench
51	EPI_ISL_1615586	Illumina NextSeq 550	custom minimap2 + iVar pipeline
52	EPI_ISL_1615587	Illumina NextSeq 550	custom minimap2 + iVar pipeline
53	EPI_ISL_1615588	Illumina NextSeq 550	custom minimap2 + iVar pipeline
54	EPI_ISL_1615589	Illumina NextSeq 550	custom minimap2 + iVar pipeline
55	EPI_ISL_1615590	Illumina NextSeq 550	custom minimap2 + iVar pipeline
56	EPI_ISL_1615600	Illumina NextSeq 550	custom minimap2 + iVar pipeline
57	EPI_ISL_1615617	Illumina NextSeq 550	custom minimap2 + iVar pipeline
58	EPI_ISL_1615620	Illumina NextSeq 550	custom minimap2 + iVar pipeline
59	EPI_ISL_1615621	Illumina NextSeq 550	custom minimap2 + iVar pipeline
60	EPI_ISL_1615656	Illumina NextSeq 550	custom minimap2 + iVar pipeline
61	EPI_ISL_1615658	Illumina NextSeq 550	custom minimap2 + iVar pipeline
62	EPI_ISL_1615660	Illumina NextSeq 550	custom minimap2 + iVar pipeline
63	EPI_ISL_1633339	illumina Miseq	CLC Genomics Workbench
64	EPI_ISL_1634442	illumina Miseq	CLC Genomics Workbench
65	EPI_ISL_1634465	illumina Miseq	CLC Genomics Workbench
66	EPI_ISL_1634467	illumina Miseq	CLC Genomics Workbench
67	EPI_ISL_1634470	illumina Miseq	CLC Genomics Workbench
68	EPI_ISL_1634471	illumina Miseq	CLC Genomics Workbench
69	EPI_ISL_1634474	illumina Miseq	CLC Genomics Workbench
70	EPI_ISL_1634475	illumina Miseq	CLC Genomics Workbench
71	EPI_ISL_1634477	illumina Miseq	CLC Genomics Workbench
72	EPI_ISL_1660412	illumina Miseq	CLC Genomics Workbench
73	EPI_ISL_1660413	illumina Miseq	CLC Genomics Workbench
74	EPI_ISL_1660414	illumina Miseq	CLC Genomics Workbench
75	EPI_ISL_1660415	illumina Miseq	CLC Genomics Workbench
76	EPI_ISL_1660416	Illumina MiSeq	CLC Genomics Workbench
77	EPI_ISL_1660417	Illumina MiSeq	CLC Genomics Workbench
78	EPI_ISL_1660418	illumina Miseq	CLC Genomics Workbench
79	EPI_ISL_1660419	illumina Miseq	CLC Genomics Workbench

80	EPI_ISL_1660420	illumina Miseq	CLC Genomics Workbench
81	EPI_ISL_1660472	illumina Miseq	CLC Genomics Workbench
82	EPI_ISL_1660473	illumina Miseq	CLC Genomics Workbench
83	EPI_ISL_2709406	Illumina MiSeq	CLC Genomics Workbench
84	EPI_ISL_2709407	Illumina MiSeq	CLC Genomics Workbench
85	EPI_ISL_2709408	Illumina MiSeq	CLC Genomics Workbench
86	EPI_ISL_2709409	Illumina MiSeq	CLC Genomics Workbench
87	EPI_ISL_2709410	Illumina MiSeq	CLC Genomics Workbench
88	EPI_ISL_2709411	Illumina MiSeq	CLC Genomics Workbench
89	EPI_ISL_2709412	Illumina MiSeq	CLC Genomics Workbench
90	EPI_ISL_2709413	Illumina MiSeq	CLC Genomics Workbench
91	EPI_ISL_2709414	Illumina MiSeq	CLC Genomics Workbench
92	EPI_ISL_2709415	Illumina MiSeq	CLC Genomics Workbench
93	EPI_ISL_2709418	Illumina MiSeq	CLC Genomics Workbench
94	EPI_ISL_2709419	Illumina MiSeq	CLC Genomics Workbench
95	EPI_ISL_2709420	Illumina MiSeq	CLC Genomics Workbench
96	EPI_ISL_2709421	Illumina MiSeq	CLC Genomics Workbench
97	EPI_ISL_2709422	Illumina MiSeq	CLC Genomics Workbench
98	EPI_ISL_2709423	Illumina MiSeq	CLC Genomics Workbench
99	EPI_ISL_2709424	Illumina MiSeq	CLC Genomics Workbench
100	EPI_ISL_2709425	Illumina MiSeq	CLC Genomics Workbench
101	EPI_ISL_2709426	Illumina MiSeq	CLC Genomics Workbench
102	EPI_ISL_2709427	Illumina MiSeq	CLC Genomics Workbench
103	EPI_ISL_2709430	Illumina MiSeq	CLC Genomics Workbench
104	EPI_ISL_2709431	Illumina MiSeq	CLC Genomics Workbench
105	EPI_ISL_2709433	Illumina MiSeq	CLC Genomics Workbench
106	EPI_ISL_2709434	Illumina MiSeq	CLC Genomics Workbench
107	EPI_ISL_2709435	Illumina MiSeq	CLC Genomics Workbench
108	EPI_ISL_2709436	Illumina MiSeq	CLC Genomics Workbench
109	EPI_ISL_2709437	Illumina MiSeq	CLC Genomics Workbench
110	EPI_ISL_2709438	Illumina MiSeq	CLC Genomics Workbench
111	EPI_ISL_2709439	Illumina MiSeq	CLC Genomics Workbench
112	EPI_ISL_2709441	Illumina MiSeq	CLC Genomics Workbench
113	EPI_ISL_2709442	Illumina MiSeq	CLC Genomics Workbench
114	EPI_ISL_2709443	Illumina MiSeq	CLC Genomics Workbench
115	EPI_ISL_2709444	Illumina MiSeq	CLC Genomics Workbench
116	EPI_ISL_2709445	Illumina MiSeq	CLC Genomics Workbench
117	EPI_ISL_2918627	Illumina MiSeq	BWA-MEM
118	EPI_ISL_2918628	Illumina MiSeq	BWA-MEM
119	EPI_ISL_2918629	Illumina MiSeq	BWA-MEM
120	EPI_ISL_2918630	Illumina MiSeq	BWA-MEM

121	EPI_ISL_2918631	Illumina MiSeq	BWA-MEM
122	EPI_ISL_2918632	Illumina MiSeq	BWA-MEM
123	EPI_ISL_2918633	illumina MiSeq	CLC Genomics Workbench
124	EPI_ISL_2918634	illumina MiSeq	CLC Genomics Workbench
125	EPI_ISL_2918635	illumina MiSeq	CLC Genomics Workbench
126	EPI_ISL_2918636	illumina MiSeq	CLC Genomics Workbench
127	EPI_ISL_2918637	illumina MiSeq	CLC Genomics Workbench
128	EPI_ISL_2918638	illumina MiSeq	CLC Genomics Workbench
129	EPI_ISL_2918640	illumina MiSeq	CLC Genomics Workbench
130	EPI_ISL_2918641	illumina MiSeq	CLC Genomics Workbench
131	EPI_ISL_2918642	illumina MiSeq	CLC Genomics Workbench
132	EPI_ISL_2918644	illumina MiSeq	CLC Genomics Workbench
133	EPI_ISL_2918645	illumina MiSeq	CLC Genomics Workbench
134	EPI_ISL_2918646	illumina MiSeq	CLC Genomics Workbench
135	EPI_ISL_2918647	illumina MiSeq	CLC Genomics Workbench
136	EPI_ISL_2918648	illumina MiSeq	CLC Genomics Workbench
137	EPI_ISL_2918649	illumina MiSeq	CLC Genomics Workbench
138	EPI_ISL_3030040	Illumina NextSeq 550	custom minimap2 + iVar pipeline
139	EPI_ISL_3030041	Illumina NextSeq 550	custom minimap2 + iVar pipeline
140	EPI_ISL_3030042	Illumina NextSeq 550	custom minimap2 + iVar pipeline
141	EPI_ISL_3030043	Illumina NextSeq 550	custom minimap2 + iVar pipeline
142	EPI_ISL_3030044	Illumina NextSeq 550	custom minimap2 + iVar pipeline
143	EPI_ISL_3030045	Illumina NextSeq 550	custom minimap2 + iVar pipeline
144	EPI_ISL_3030046	Illumina NextSeq 550	custom minimap2 + iVar pipeline
145	EPI_ISL_3030048	Illumina NextSeq 550	custom minimap2 + iVar pipeline
146	EPI_ISL_3030049	Illumina NextSeq 550	custom minimap2 + iVar pipeline
147	EPI_ISL_3030050	Illumina NextSeq 550	custom minimap2 + iVar pipeline
148	EPI_ISL_3030051	Illumina NextSeq 550	custom minimap2 + iVar pipeline
149	EPI_ISL_3030052	Illumina NextSeq 550	custom minimap2 + iVar pipeline
150	EPI_ISL_3030053	Illumina NextSeq 550	custom minimap2 + iVar pipeline
151	EPI_ISL_3030054	Illumina NextSeq 550	custom minimap2 + iVar pipeline
152	EPI_ISL_3030055	Illumina NextSeq 550	custom minimap2 + iVar pipeline
153	EPI_ISL_3030056	Illumina NextSeq 550	custom minimap2 + iVar pipeline
154	EPI_ISL_3030057	Illumina NextSeq 550	custom minimap2 + iVar pipeline
155	EPI_ISL_3030058	Illumina NextSeq 550	custom minimap2 + iVar pipeline
156	EPI_ISL_3030059	Illumina NextSeq 550	custom minimap2 + iVar pipeline
157	EPI_ISL_745033	Illumina MiSeq	UGENE v.37
158	EPI_ISL_747235	Illumina MiSeq	UGENE v.37
159	EPI_ISL_747236	Oxford Nanopore	
160	EPI_ISL_747238	Oxford Nanopore	
161	EPI_ISL_747239	Illumina MiSeq	UGENE v.37

162	EPI_ISL_747240	Illumina MiSeq	UGENE v.37
163	EPI_ISL_747241	Illumina MiSeq	Dragen RNA Pathogen Detection 3.5.14

Supplementary Table S2. Hotspot mutations (frequency >10% of samples) found in SARS-CoV-2 genome sequences circulating in Indonesia. Mutations are sorted in descending frequency order.

**No.**	**Gene_Mutation**	**Frequency (%)***	**No.**	**Gene_Mutation**	**Frequency (%)***
1	S_D614G	100.00	21	S_V1264L	39.26
2	NSP12_P323L	100.00	22	ORF7b_T40I	39.26
3	S_P681R	67.48	23	NSP6_T77A	39.26
4	ORF3a_Q57H	49.08	24	NSP4_V167L	39.26
5	N_D377Y	43.56	25	NSP4_T492I	39.26
6	S_T478K	42.33	26	NSP3_T678I	39.26
7	S_T19R	42.33	27	NSP3_P822L	39.26
8	S_R158del	42.33	28	NSP3_P1469S	39.26
9	S_L452R	42.33	29	NSP3_P1228L	39.26
10	S_F157del	42.33	30	NSP3_A488S	39.26
11	S_E156G	42.33	31	NSP2_P200L	39.26
12	ORF7a_T120I	42.33	32	NSP14_A394V	39.26
13	ORF3a_S26L	42.33	33	N_G215C	39.26
14	NSP13_P77L	42.33	34	S_N439K	34.36
15	NSP12_G671S	42.33	35	NSP3_T350I	30.06
16	N_R203M	42.33	36	N_D63G	26.99
17	M_I82T	42.33	37	NSP3_S126L	25.77
18	S_D950N	41.72	38	NSP13_S259L	25.77
19	ORF7a_V82A	41.72	39	NSP6_L75F	20.25
20	N_T205I	41.72	40	S_G142D	11.04

Supplementary Table S3. Analysis of mutations found in dominant variants (B.1.466.2, B.1.470 and B.1.617.2) on protein stability. Stability change is shown as ΔGmutant – ΔGwildtype. (ΔΔG > 0.5 : destabilize; ΔΔG < -0.5 : stabilize; -0.5 < ΔΔG < 0.5 : neutral). Mutations in grey are mutations appearing with a prevalence of higher than 90% in each variant (not appearing in all sequences).

Variant	Mutation	ΔΔG Protein (kcal/mol)	Result
B.1.466.2	S_D614G	-0.55	Stabilize
	S_N439K	-0.97	Stabilize
	S_N439K/D614G	-1.52	Stabilize
	N_T205I	0.02	Neutral
	NSP3_P822L	0.14	Neutral
	NSP12_P323L	0.86	Destabilize
	ORF3a_Q57H	0.55	Destabilize
B.1.470	S_D614G	-0.55	Stabilize
	NSP12_P227L	1.25	Destabilize
	NSP12_P323L	0.86	Destabilize
	NSP12_P227L/P323L	2.01	Destabilize
	ORF3a_Q57H	0.55	Destabilize
B.1.617.2	S_T19R	-0.77	Stabilize
	S_E156G	3.04	Destabilize
	S_L452R	0.98	Destabilize
	S_T478K	-0.56	Stabilize
	S_D614G	-0.55	Stabilize
	S_T19R/E156G/L452R/T478K/D614G	1.77	Destabilize
	N_R203M	0.32	Neutral
	N_D377Y	-1.39	Stabilize
	N_R203M/D377Y	-1.02	Stabilize
	M_I82T	0.56	Destabilize
	NSP3_T678I	2.65	Destabilize
	NSP3_P1469S	4.98	Destabilize
	NSP3_P1228L	1.48	Destabilize
	NSP3_A488S	-0.06	Neutral
	NSP3_T678I/P1469S/P1228L/A488S	9.15	Destabilize
	NSP12_P323L	0.86	Destabilize
	NSP12_G671S	1.01	Destabilize
	NSP12_P323L/G671S	1.86	Destabilize
	NSP13_P77L	1.85	Destabilize
	ORF3a_S26L	0.7	Destabilize
	ORF7a_T120I	0.38	Neutral

Supplementary Table S4. Molecular docking analysis of spike protein (wildtype, B.1.466.2, B.1.470 and B.1.617.2 variant) with ACE2 receptor.

Protein Variant	Docking Score (a.u)	Binding Affinity (kcal/mol)
S_wild type	-112.4	-10.0
S_B.1.466.2	-106.5	-10.1
S_B.1.470	-110.4	-10.6
S_B.1.617.2	-109.5	-12.2
